# Improved Copper–Epoxy Adhesion by Laser Micro- and Nano-Structuring of Copper Surface for Thermal Applications

**DOI:** 10.3390/polym13111721

**Published:** 2021-05-24

**Authors:** Mario Mora, Hippolyte Amaveda, Luis Porta-Velilla, Germán F. de la Fuente, Elena Martínez, Luis A. Angurel

**Affiliations:** Instituto de Nanociencia y Materiales de Aragón (INMA), CSIC—Universidad de Zaragoza, 50018 Zaragoza, Spain; mmora@unizar.es (M.M.); hippo@unizar.es (H.A.); porveli@unizar.es (L.P.-V.); german.delafuente.leis@csic.es (G.F.d.l.F.); angurel@unizar.es (L.A.A.)

**Keywords:** adhesion, mechanical strength, epoxy, laser, surface nano-structuring, thermal conductivity

## Abstract

The objective of this work is the enhancement of metal-to-metal bonding to provide high thermal conductivity together with electrical insulation, to be used as heat sinks at room and cryogenic temperatures. High thermal conductive metal (copper) and epoxy resin (Stycast 2850FT) were used in this study, with the latter also providing the required electrical insulation. The copper surface was irradiated with laser to induce micro- and nano-patterned structures that result in an improvement of the adhesion between the epoxy and the copper. Thus, copper-to-copper bonding strength was characterized by means of mechanical tensile shear tests. The effect of the laser processing on the thermal conductivity properties of the Cu/epoxy/Cu joint at different temperatures, from 10 to 300 K, is also reported. Using adequate laser parameters, it is possible to obtain high bonding strength values limited by cohesive epoxy fracture, together with good thermal conductivity at ambient and cryogenic temperatures.

## 1. Introduction

The adhesion between epoxy resin and metals is of great relevance in diverse industry sectors, such as automotive, aerospace, energy, and electronics [1,2,3]. Particularly, the fabrication of metal/epoxy/metal joints and metal/epoxy coatings usually requires a previous treatment of the metal surface in order to achieve good adhesion strength. Wet-chemical processes have often been used with this aim. In the case of copper, these consist of removing first the native oxides and organic contaminants (by immersion in concentrated hydrochloric acid solution, for example) and then exposing the surface to some oxidation agents (for example alkaline solutions, such as sodium chlorite and sodium hydroxide solution) at temperatures of 70–95 °C during a few minutes [4,5]. This method, widely used in electronics, results in the formation of the so-called black and red oxides on the metal surface. The resulting improvement of the copper/epoxy adhesion strength has been attributed to the nucleation of acicular CuO crystals on top of the Cu_2_O layer [4]. However, the Cu/epoxy bonds prepared using this protocol present some drawbacks, such as their poor durability in acidic environments [1,4] due to corrosion of the oxide, causing delamination problems. The use of aggressive agents in chemical treatments may create a large volume of hazardous and contaminant wastes that can negatively affect natural environment and human health. 

Laser irradiation of metallic surfaces has gained attention in recent years as a clean and environmentally friendly tool that can effectively produce significant changes in the surface wettability [6,7,8,9,10,11,12,13] and in the adhesion of metal/epoxy joints [3,14,15,16,17]. Adhesion between polymers and metals involves different mechanisms such as interlocking, adsorption, and chemical bonding, among others [2]. Thus, increasing surface roughness may improve the adhesion strength, but not only, as effective wetting to provide good contact between the two components in the bond interface is essential. Laser treatments affect surface chemistry and surface topography, with the formation of micro- and nano-patterned structures, thus facilitating changes in wettability and metal/epoxy adhesion properties. Surface extreme wetting behavior after laser irradiation has been observed for aluminum [7,11,12], copper [7,8,9], titanium [10], steel [6,13], and other alloys [6,9]. An evolution in time of the wetting behavior, which is usually analyzed by measuring the time dependence of the static contact angle (SCA), has been reported by several groups [6,7,8,9]. Particularly, a transition from initial (super)hydrophilicity (after laser processing) to a stable (super)hydrophobicity state has been observed, thus indicating the relevance of surface chemistry in this process. The SCA vs. time curve depends on the laser processing conditions and on the irradiated metal, with the whole transition taking place in periods that range from several days to several weeks under ambient conditions. The adsorption of organic compounds present in the surrounding air is believed to play a crucial role on the observed wettability evolution after laser treatments [10,11]. However, in the case of copper, the role of the CuO de-oxidation in this process is still a subject of controversy [9,18]. Some authors have proposed different treatments at low temperatures after laser processing either to accelerate this transition [13] or to retain [12] or recover [11] the wetting conditions in a number of metals.

The adhesion strength of metal/epoxy bonds can be deteriorated by mechanical stresses during operation, which can be diverse depending on the application. In the particular case of superconducting coils, mechanical stresses are normally due to thermal cycling and to electromagnetic Lorentz forces. In the first case, these are caused by the different thermal contraction coefficients of the joint components when cooling the device from room temperature (RT) to cryogenic temperatures. On the other hand, Lorentz forces, which are caused by the application of large currents in the presence of high magnetic fields, could also produce additional stresses in the bond interfaces.

In previous work [19], which is focused on the thermal and electromagnetic behavior of a superconducting coil, we found unreliable behavior of a copper/epoxy joint used in that system. This type of joint was used to connect thermally the superconducting coil to a copper support plate, which was, in turn, in thermal contact with the second stage of a cryocooler (conduction-cooled coil). The copper/epoxy bond failure, observed after several thermal cycles from RT to cryogenic temperatures, resulted in a poor thermal conductance between the copper support and the coil. The epoxy used, Stycast 2850FT (with catalyst 9), is an electrical insulator that contains embedded Al_2_O_3_ (alumina) particles to increase its thermal conductivity. This epoxy has been used in cryogenic applications and has a total linear contraction from RT to 77 K (*L*_293K_ − *L*_77K_)/*L*_293K_ = 0.4%, which is larger than for copper, 0.3% [20].

The aim of this work is to improve the copper/epoxy bond strength and to achieve more reliable adhesion behavior without using chemical oxidizing agents. Several laser treatments were performed on the copper surface using a near-infrared laser and pulse width ranging between 10 and 120 ns. The effect of the different irradiation parameters (i.e., fluence, irradiance, and thermal incubation) on the Cu surface wettability and on the Cu/epoxy/Cu bond strength has been studied. Moreover, the effect of thermal cycling of the Cu/epoxy/Cu joint on its mechanical properties, along with its thermal conductivity at different temperatures (*T*) from 10 K to 300 K, are analyzed.

## 2. Materials and Methods

### 2.1. Materials

Epoxy resin Loctite Stycast 2850FT (Henkel, Düsseldorf, Germany) was used to make the joints between two copper plates, using the catalysts 9 or 23LV. The viscosity of the epoxy is 58 and 5.6 kPa·s for catalyst 9 and 23LV, respectively. The curing time at 25 °C is 16–24 h for both catalysts, whereas the pot life is larger for the latter (60 vs. 45 min). This epoxy has a thermal conductivity *ĸ_ep_* ≈ 1 W m^−1^ K^−1^ at room temperature (RT) [21], which decreases to ≈0.64 W m^−1^ K^−1^ at *T* = 70 K [22]. Commercial copper plates (from Sanmetal, Zaragoza, Spain) of 99.9% purity and average roughness of 0.45 ± 0.11 μm were used. The residual–resistance ratio (RRR), which is usually defined as the ratio between the electrical resistance at 293 K and at 4.2 K, is about 100. The *ĸ_Cu_*(*T*) dependence presents a peak at *T* ≈ 25 K, having values of *ĸ_Cu_* ≈ 397 and 602 W m^−1^ K^−1^ at 295 K and 70 K, respectively [20,23,24]. Note that the purer the material, the higher its RRR and its thermal conductivity values at cryogenic temperatures [20,23].

### 2.2. Laser Irradiation and Surface Characterization

The copper surfaces were first cleaned to remove organic contaminants by the following process: they were washed with soap and rinsed with water; then, they were subjected to 15 min in an ultrasonic bath with acetone. After this treatment, the samples were rinsed with distilled water and finally dried at 60 °C in air. Later, the copper surfaces were irradiated with an Ytterbium-pulsed fiber laser (model PEDB-400B, Perfect Laser, Co., Ltd., Wuhan, China), with central wavelength *λ* = 1060–1070 nm, under different pulse repetition frequencies (*f_rep_*) and pulse width (*τ_p_*) values. The focusing of the laser beam was realized by a flat field scanning lens system (effective focal length of 163 mm), resulting in a circular beam of 1/e^2^ diameter 2*r_b_* = 65 μm, which was determined in the sample processing plane using the *D*^2^-method proposed by Liu [25].

Two different laser processing modes were used: (*i*) In the first method, called laser line scanning mode (LSM), the laser beam moves in a given direction at a steering speed *v*_b_, scanning a stationary line of fixed length (30 mm in our case), while the sample is moving in the orthogonal direction at a velocity vs. [26]. In this work, constant values of *f_rep_* = 900 kHz, *τ_p_* = 10 ns, *v*_b_ = 1 m/s, and vs. = 1 m/h were used, whereas the pulse fluence (*F*_p_) was changed from 0.70 to 2.35 J/cm^2^ (by varying the laser average power, *P* from 21 to 70 W). These values correspond to irradiance (*I*) values between 70 and 235 MW/cm^2^, respectively. (*ii*) In the second method, called bit-map, instead of irradiating the whole surface, a square grid of “pixels” is drawn by the laser. Three different distances between the centers of consecutive pixels were used, 169, 84.5, and 42.3 μm, corresponding to 150, 300, and 600 dpi (dots per inch), respectively. To form each pixel, the laser irradiates at that location using the burst mode, which consists in using sequences (or bursts) of laser pulses. In this case, 25 pulses per burst were used, with *f_rep_* = 50 kHz, *τ_p_* = 120 ns, *F*_p_ = 9.19 J/cm^2^, and *I* = 77 MW/cm^2^ and *P* = 15.3 W. All laser treatments were performed in air. 

Microstructural characterization was performed by a MERLIN field-emission scanning electron microscope (FE-SEM) (Carl Zeiss, Jena, Germany), equipped with an energy dispersive X-ray spectroscopy (EDS) system (Oxford Instruments, Abingdon, UK). The surface roughness was analyzed by confocal microscopy (Plμ-200, SENSOFAR, Barcelona, Spain). The wettability of non-treated and laser-irradiated surfaces was analyzed by measuring the static contact angle using a micropipette (model Multipette^®^ E3x, Eppendorf AG, Hamburg, Germany), a digital camera (model UI-3080CP Rev.2, IDS, Obersulm, Germany), and the ImageJ software with installed LBADSA (Low Bond Axisymmetric Drop Shape Analysis) plugin [27]. SCA was measured using a 6 μL droplet of water or epoxy after waiting for 1 min for stabilization.

### 2.3. Joint Manufacturing Process and Mechanical Characterization 

After treating both copper surfaces of the joint with the same conditions, they were epoxy impregnated by the deep coating method and cured for 24 h at 25 °C under a small pressure of about 1 kPa. A mold accommodating ten lap joints was used for better control of the overlap length and pressure homogeneity during the curing process. In this work, most joints were prepared just after laser processing of the copper surfaces (in the time interval typically within 5–15 min). Moreover, in order to analyze the effect of the time between the laser treatment and the joint manufacture, some joints were prepared 22 days after laser processing (stored in air and at ambient temperature). Note that unless otherwise indicated, the epoxy was prepared using catalyst 23LV, since most joints and experiments were performed using this catalyst for the reasons given below.

**Figure 1:**
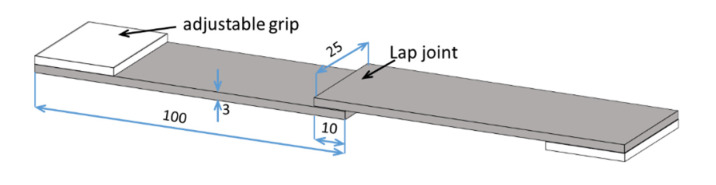
Schematics of the copper–epoxy–copper single lap joint prepared for the tensile shear test. Dimensions are given in mm.

Lap tensile shear strength tests were performed to evaluate the bonding strength of the Cu/epoxy/Cu joints using a universal mechanical testing machine (model HS-10-S, capacity 100 kN, Hoytom, Leioa, Spain). Figure 1 shows a scheme of the lap joints prepared for mechanical characterization. The dimensions of the specimens (Cu plates: 100 mm × 25 mm × 3 mm; lap joint: 25 mm × 10 mm) and the test displacement rate (1.0 mm/min) were chosen according to the Standard Test Method for Apparent Shear Strength of Single-Lap-Joint Adhesively Bonded Metal Specimens by Tension Loading (Metal-to-Metal) (ASTM D1002-01). The number of specimens for each testing condition was at least two. The bond strength was calculated as the failure load divided by the joint area (250 mm^2^). After each mechanical experiment, the failure mechanism was analyzed by photographing the surfaces of both parts.

Some joints were also prepared with non-irradiated copper, as a reference. These joints were made either using as-received Cu plates (after cleaning to remove organic contaminants) or with polished plates using 1200 grit sandpaper (and then cleaned). These types of reference joints will be named “as-received” and “polished”, respectively. 

Finally, in order to compare laser processing vs. chemical treatment on the mechanical behavior of lap joints, some of them were prepared after having treated the copper surfaces with a chemical oxidizing agent, based on NaClO_2_ at a temperature of 85 °C during 3 or 5 min, with the same procedure described in [4]. 

The effect of thermal cycling on the strength of selected joints was also analyzed by testing nominally identical joints after 10 thermal cycles. Each thermal cycle starts from the specimen at RT; it is cooled down at 77 K by fully immersing the sample in liquid nitrogen (LN) and waiting for thermal stabilization, and then back to RT. Each thermal cycle takes about 6–7 min. More in detail, one minute after the joints are immersed in LN, they reach 77 K. Then, they are kept at this temperature for about one minute and finally removed from the LN bath and warmed up during 4–5 min to achieve RT. 

### 2.4. Thermal Characterization of the Joints at Cryogenic Temperatures

**Figure 2:**
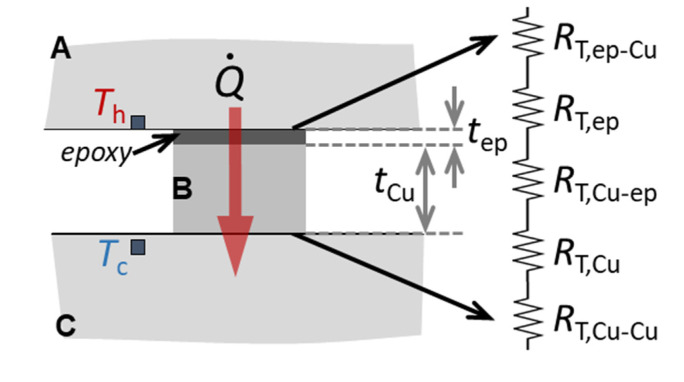
Scheme (cross-section view, not to scale) of the experiment to characterize the thermal resistivity of a Cu/epoxy/Cu joint. A, B, and C are made of copper (A and B are joined by 2850FT epoxy). The measured thermal resistance of the joint is the contribution of different thermal resistances, as explained in the text. The temperature difference across the joint (*T_h_* − *T_c_*) is measured by two thermometers. The contact area of the joint is *A_J_* = 2 cm^2^, *t_Cu_* = 6 mm and *t_ep_* ≈ 60–80 μm.

The thermal resistance of different Cu/epoxy/Cu joints was characterized using the set-up presented in Figure 2. Copper parts named A and B in the figure were glued together by 2850FT epoxy after both surfaces of the joint were treated as explained above. The contact area of the joint is *A_J_* = 2 cm^2^, and the thickness of plate B and epoxy layer is *t_Cu_* = 6 mm and *t_ep_* ≈ 60–80 μm, respectively. Part A is the heat source and has a heater of resistance 50 Ω that provides a constant heat flow across the joint, $\dot{Q}$, which can be varied. Copper plate C is the heat sink (or cold end) and is thermally anchored to the second stage of a cryocooler. It has another heater, which is used to control the temperature of the joint from 10 to 77 K, by means of a temperature controller (LakeShore Cryotronics, Westerviller, OH, USA). The joint is inside a cryostat under high vacuum conditions (about 10^−5^ hPa). In each experiment run, after the set temperature becomes stable, a constant $\dot{Q}$ value is applied across the joint. Temperatures *T_h_* and *T_c_* eventually reach a stable value, and the measured difference between both at the equilibrium, (*T_h_* − *T_c_*)*_eq_*, can be used to estimate the thermal resistance of the joint at each temperature, *R_T,J_*(*T*). The latter is the sum of different contributions:

(1) $$R_{T,J}\left(T\right)\approx \frac{{\left(T_{h}-T_{c}\right)}_{eq}}{\dot{Q}}\approx R_{T,ep-Cu}+R_{T,ep}+R_{T,Cu-ep}+R_{T,Cu}+R_{T,Cu-Cu}\;\left[K/W\right].\;$$

Here, *R_T,ep_* and *R_T,Cu_* are the epoxy and copper bulk components of the thermal resistance, which depend on temperature. For small increments of the temperature during each experiment and by assuming a one-dimensional model approximation, these values can be calculated as:

(2) $$R_{T,ep}\left(T\right)\approx \frac{1}{\kappa_{ep}\left(T\right)}\frac{t_{ep}}{A_{J}};\;R_{T,Cu}\left(T\right)\approx \frac{1}{\kappa_{Cu}\left(T\right)}\frac{t_{Cu}}{A_{J}}.\;$$

The other components in Equation (1), which are also temperature dependent, correspond to the thermal contact resistance contributions across the different surface interfaces: epoxy/copper (*R_T,ep_*_−*Cu*_ and *R_T,Cu−ep_*, which are assumed equivalent) and copper/copper (*R_T,Cu−Cu_*). In order to analyze if the epoxy/copper thermal contact resistance contributes significantly to *R_T,J_*, a similar set-up as in Figure 2 but without epoxy was also characterized. Thus, the value of *R_T,Cu−Cu_* may be estimated using the measured value *R_T,J−_*_REF_, given by:

(3) $$R_{T,J-\mathrm{REF}}\left(T\right)\approx \frac{{\left(T_{h}-T_{c}\right)}_{eq}}{\dot{Q}}\approx R_{T,Cu}+2R_{T,Cu-Cu}\;\left[K/W\right].\;$$

From these two measurements, it is possible to estimate all contributions to the overall thermal resistance of the joint for each temperature. 

## 3. Results and Discussion

### 3.1. Laser-Induced Copper Surface Modifications 

**Figure 3:**
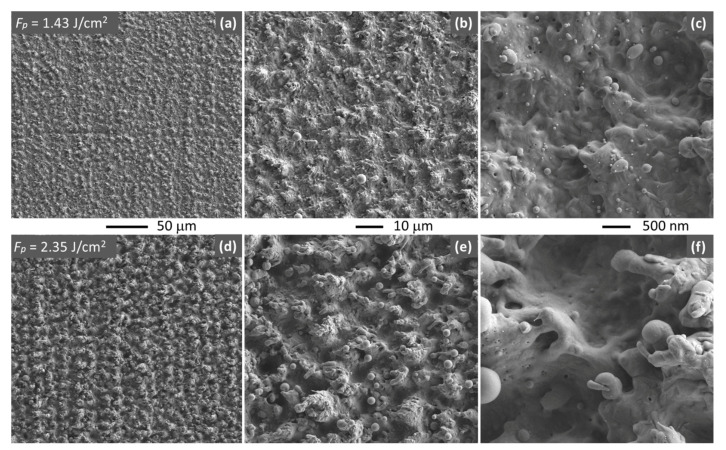
SEM images (secondary electrons) of the copper surfaces processed by laser with LSM at two different pulse fluences: (**a**–**c**) *F*_p_ = 1.43 J/cm^2^, (**d**–**f**) *F*_p_ = 2.35 J/cm^2^, with different magnification values.

Figure 3 shows the Cu surface topography after laser processing using laser scanning method with two different pulse fluence values. The images clearly show that processing with increasing pulse laser fluence values increases the surface roughness and the amount of melted material. Some spherical particles are observed in both cases, although these are larger with increasing fluence. With this processing method, all the surface is processed, resulting in a homogenous surface microstructure. 

The average roughness, Ra, of the LSM processed copper surfaces was measured at different 256 μm × 191 μm areas using confocal microscopy. A relevant increment of Ra from 0.68 to 2.89 μm was observed upon increasing *F*_p_ from 1.43 to 2.35 J/cm^2^ (Ra standard deviations 0.12–0.19 μm). The non-irradiated copper surfaces have typical roughness of 0.45 ± 0.11 μm (as-received) and 0.20 ± 0.05 μm (polished with 1200 sandpaper).

**Figure 4:**
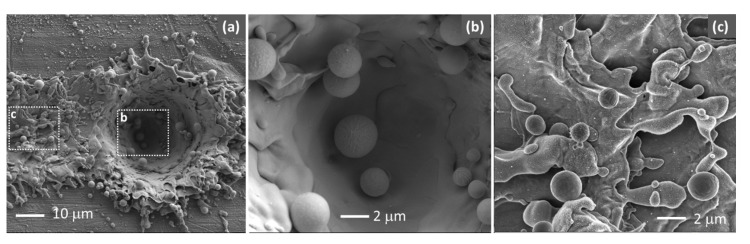
(**a**) SEM image of the copper surface laser-processed with bit-map method (300 dpi) around a “pixel”. Images (**b**,**c**) are selected areas from (**a**) using a higher magnification. Images (**a**,**b**) correspond to secondary electrons, and (**c**) corresponds to Inlens detector.

Figure 4 shows SEM images of copper surface after being processed with the bit-map method (300 dpi). With this laser processing procedure, each pixel is characterized by a hole that is surrounded by a large amount of molten material. The presence of numerous round particles observed on the processed surface indicates that melting is the main mechanism in this process, as expected from the long pulse width (120 ns) used. It is also observed that not only the area corresponding to a pixel is processed by the laser but a trail near each pixel in the direction of laser beam movement has also been generated (see the left-hand part of Figure 4a). This is due to a certain delay between the systems that control the laser switching (on/off) and the galvo mirror movement. The treated area of each pixel is around 40–45 μm, so that all the surface is treated with 600 dpi, but not in the case of 150 and 300 dpi (as observed in the image of Figure 4a).

**Figure 5:**
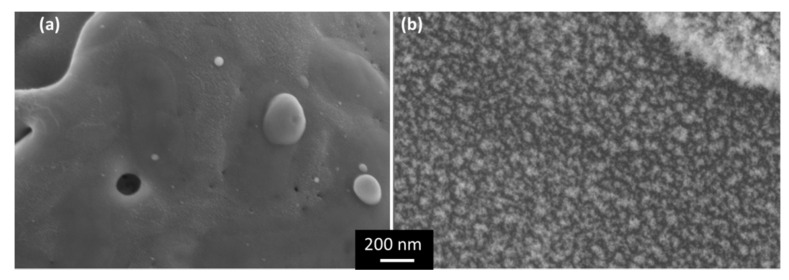
SEM images (Inlens detector) of copper surfaces processed with (**a**) LSM, (**b**) bit-map method, just after laser processing. The same magnification was used in both images.

Figure 5 shows a detail of the surface characteristics of the Cu surfaces after each of the two types of laser processes. In the case of the sample processed using the LSM mode (Figure 5a), the surface has the typical aspect of a molten surface. In the case of the bit-map surface, a similar aspect was observed in the center of each pixel, but in the surface of the material that has been projected in the ablation process, with higher oxygen content values, a fuzz type nanostructure has been generated (Figure 5b). 

### 3.2. Time Evolution of Surface Characteristics after Laser Processing

Topography, as well as micro- and nanoscale patterning characteristics of laser processed copper surfaces, as described in the previous section, remain almost invariant over time in the interval of 22 days of this study. However, chemical properties can evolve significantly with time and could thus affect wettability and adhesion properties. Several measurements have been performed to analyze these effects. 

EDS analysis was performed on the different laser-irradiated surfaces in order to analyze the time evolution of the surface oxygen content after laser processing. The results are collected in Table 1. Note that due to EDS information depth and the irregular rough surfaces, the absolute percentage values must be taken with caution. Table 1 collects the oxygen content obtained by EDS analysis of copper surfaces—just after laser processing and 22 days later—for the various laser processing methods and parameters applied. The values correspond to average values within an approximate area of 250 μm × 370 μm. The oxygen content increases with pulse fluence (in case of LSM) or with dpi value (for bit-map method). In the former laser treatment type, the oxygen content barely changes, although in some cases, it is found to decrease as a function of time. The latter has been observed by other groups after laser processing [9], and it has been attributed to the partial reduction of CuO into more stable Cu_2_O. On the contrary, in the case of the bit-map process, an appreciable increase of oxygen content with time has been observed for all samples, irrespective of the used dpi value. It must be noted that these samples exhibit very inhomogeneous behavior, inherent to the applied method, with much lower oxygen content present within the pixels and higher around each pixel.

The wettability of water (apparent contact angle) on copper surfaces varies strongly over time as gathered from Table 1. This has been attributed partially to the above-mentioned partial deoxidation of the uppermost layer of the CuO (hydrophilic) into Cu_2_O (hydrophobic) [7,9], and to the increase in carbon content on the surface due to the adsorption of organic molecules from the ambient atmosphere [7]. On the contrary, the contact angle of epoxy remains constant over time, at least up to 22 days after laser processing analyzed in the present study. 

### 3.3. Microstructural Characteristics of Cu/Epoxy/Cu Joints 

**Figure 6:**
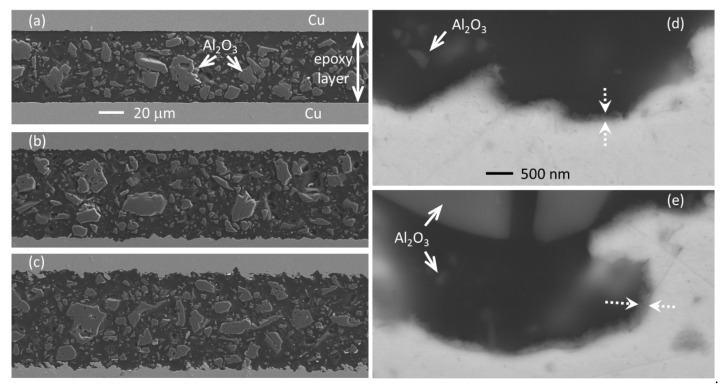
**Left column**: SEM images (secondary electrons) of the cross-sections of the three Cu/epoxy/Cu joints that were cut and polished after thermal measurements: (**a**) non-irradiated copper surfaces; (**b,c**) laser processed surfaces using LSM with *F*_p_ = 1.43 and 2.35 J/cm^2^, respectively (same magnification in **a**,**b**,**c**). **Right column**: (**d,e**) SEM images (backscattered electrons) of the interface between Cu and epoxy for the samples shown in (**b**,**c**), respectively. The same magnification was used in (**d**,**e**). Al_2_O_3_ particles embedded in the epoxy layer are clearly seen in all images.

The full cross-sections of different Cu/epoxy/Cu joints are displayed in Figure 6. The images correspond to polished cuts of three different joints after measuring their thermal resistance properties at low temperatures. Figure 6a shows a joint with non-irradiated copper surfaces polished with 1200 grit sandpaper. Figure 6b,c correspond to joints after laser processing of Cu surfaces using LSM and *F*_p_ = 1.43, 2.35 J/cm^2^, respectively. The increase of the roughness with laser processing is clearly observed, with the epoxy following the surface of the copper in all cases (air bubbles are not observed at the Cu/epoxy interface in any case). It is worth noting that the size of the embedded Al_2_O_3_ particles in the epoxy varies from hundreds of nanometers to 20–40 μm. The thickness of the epoxy layer was measured using several images of the cross-sections, giving *t_ep_* ≈ 60, 74, and 80 μm for the three shown joints, respectively (the standard deviation is ≈4 μm in all cases). 

Figure 6d,e shows a detail of the copper/epoxy interface for the joints shown in Figure 6b,c, respectively, using higher magnification and backscattered electrons. Both images reveal good adhesion between metal and epoxy. The oxide layer in the epoxy/copper interface can be associated with the darker zones pointed by dotted arrows in Figure 6d,e. This layer exhibits a non-homogenous thickness, and it is more visible for the sample irradiated at higher intensity (Figure 6e).

### 3.4. Mechanical Bonding Strength of Cu/Epoxy/Cu Joints 

Following the procedure described in Section 2.3, we first analyzed the bond strength of the Cu/epoxy/Cu bonds for the case of non-treated surfaces with laser. With this purpose, several specimens were prepared using catalyst 9 or 23LV (10 each). The obtained bond strength values were 3.8 ± 1.3 MPa and 17.0 ± 1.3 MPa, respectively. In both cases, adhesive failure behavior was observed, which is characterized by interfacial debonding. Due to the poorest behavior obtained with catalyst 9, catalyst 23LV was selected to analyze the effect of laser processing of copper on the mechanical properties of the joints. Note that this catalyst also has important advantages associated with its lower viscosity and longer pot life.

**Figure 7:**
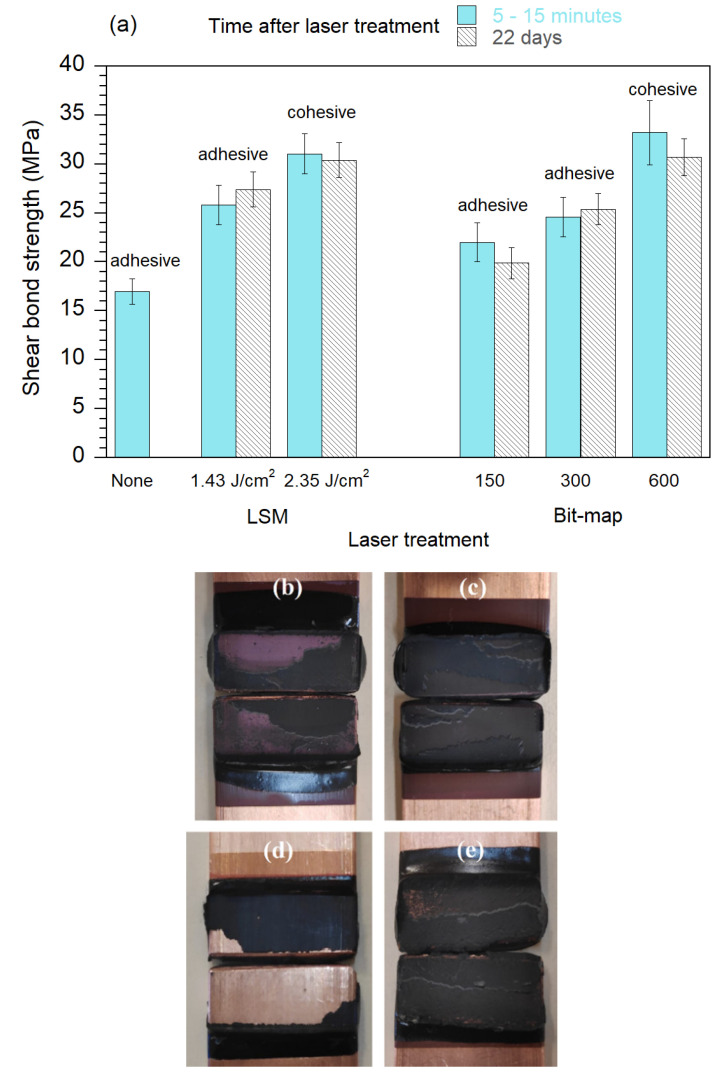
(**a**) Shear bond strength values for different joints after laser processing the copper surfaces in air using LSM or bit-map at different *F*_p_ or pixels, respectively. The value for the joint prepared with non-irradiated polished Cu is also included. The type of bond failure for each case is indicated. Pictures of some of the joints after testing: (**b**) LSM 1.43 J/cm^2^, (**c**) LSM 2.35 J/cm^2^, (**d**) bit-map 150, and (**e**) bit-map 600.

Figure 7a shows the shear bond strength of joints prepared with different laser processing conditions. The result obtained for a non-irradiated (polished) copper joint is also displayed for comparison. For both used laser processing methods, LSM and bit-map, the bond strength increases considerably upon increasing pulse fluence and number of pixels, respectively, observing a change from adhesive to cohesive failure, with measured maximum values of about 31–33 ± 2 MPa. Figure 7 also shows pictures of some of the mechanically tested joints, depicting adhesive (Figure 7b,d) and cohesive (Figure 7c,e) bond failure types. In the former type of failure, copper surface is clearly seen in the fracture images, whereas in the latter, the observed epoxy fracture would indicate that the mechanical properties of the epoxy, nor the Cu/epoxy adhesion, are the limiting factors of the joint. Cohesive failure is preferable not only because of its higher bonding strength but also because of its better reliability. It is also observed that the joints prepared 22 days after laser irradiation (samples stored in air and ambient temperature) have very similar bond strength values than their corresponding joints prepared just minutes after laser treatment. Thus, it indicates that the time elapsed between the laser treatment and the joint manufacture does not have a significant effect on the bond strength, despite the large changes on the (water) wettability surfaces observed during this period of time (Section 3.2). 

As mentioned above, both used processing methods are able to improve bond strength values and to achieve the foreseen cohesive failure behavior. However, the processing velocity for the bit-map method with 600 dpi is considerably smaller than for the LSM method (about 3.09 vs. 8.33 mm^2^/s, respectively); thus, the latter method is considered much more interesting for this practical application.

**Figure 8:**
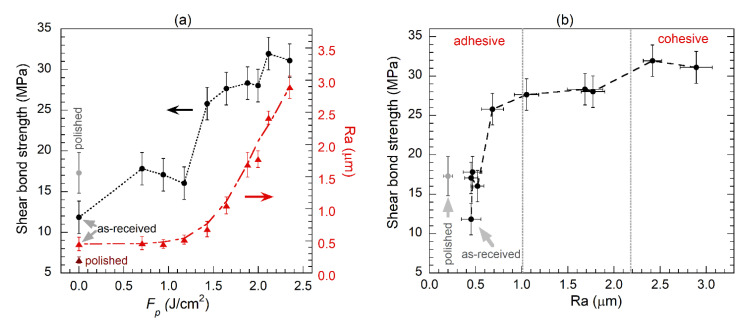
(**a**) Shear bond strength (circles) of the Cu/epoxy/Cu and Cu surface average roughness (triangles) as a function of the pulse fluence for LSM processing. The two represented values for non-irradiated samples correspond to as-received and polished surfaces, as indicated. (**b**) Shear bond strength as a function of Ra (same data as in figure (**a**)).

The relationship between the shear bond strength of Cu/epoxy/Cu bonds and the copper surface roughness induced by the laser scanning method is plotted in Figure 8. It must be noted that the joints made with as-received copper (cleaned, but not polished) have the lowest shear bond strength values (≈12 MPa). This would be attributed to the presence of native oxides, which are responsible of the very weak copper/epoxy bonding characteristics [4]. As seen in Figure 8a, laser irradiation with low pulse fluence values, up to about *F*_p_ = 1.4 J/cm^2^, produces a small increment of roughness and a small increase of the bond strength to about 16–18 MPa. Similar values are obtained for joints made with non-irradiated polished surfaces. For higher *F*_p_ values, the increase in Ra is more relevant. Note that an important increment in the shear bond strength (from values lower than 18 MPa to higher than 25 MPa) coincides with the change of tendency observed for Ra.

Figure 8b shows the dependence of the shear bond strength as a function of Ra. The increase of bond strength with roughness is sharp up to Ra < 1 μm, but it barely changes for Ra in the range of 1–2 μm. The highest values, 31–32 MPa, are obtained for 2.0 μm < Ra < 3 μm, where a fully cohesive bond failure is observed. It is worth noticing that in the cases of Ra = 1–2 μm, the bond failure is predominantly cohesive, but small areas of the joint may still have some adhesive failure characteristics, resulting in bond strength values slightly lower (≈28 MPa). This behavior could be attributed to the role of the laser-formed oxide layer, more uniform and thicker for the cases associated to the highest bond strength values, as seen in Figure 6e. The relevance of copper oxides and hydroxides formed by laser processing has been previously reported by Hernandez et al. [16], who observed an improvement of the T-peel mechanical behavior of CuZn40/epoxy joints in case of laser irradiated samples, compared to sanded surfaces. It is expected that a further increase of Ra above 3 μm could not produce any corresponding improvement of the copper/epoxy bond, as the bond failure for Ra > 2.0 μm is entirely cohesive. On the contrary, a further increase of roughness could even result in a decrease of the bond strength, as it was observed by Romoli et al. [17] for aluminium/epoxy joints. This change of behavior occurs at Ra > 3.2 μm in their study, and it was attributed to the entrapment of air bubbles within surface asperities, growing in size and number with increasing Ra. It is also important to bear in mind that despite the uniform applied pressure to the joint during curing, it is not possible to guarantee an exact uniformity of the adhesive layer thickness among all tested joints. The effect of the adhesive thickness on bond strength, which would depend on the epoxy mechanical characteristics and on the type of joint, is complex and not yet fully understood [28,29,30]. Large variations on the adhesive thickness may even produce changes on the cohesive/adhesive failure mode [28], although this is not expected to occur in the present work, given the above-mentioned thickness range measured in selected joints of this study. 

The effect of thermal cycling has been analyzed for the cases of joints made from non-irradiated and laser irradiated copper surfaces. In the former (polished surface), the shear bond strength values were 17.0 ± 1.3 MPa (without thermal cycling) and 16.8 ± 1.5 MPa (after thermal cycling). For joints made with laser-irradiated copper (LSM at *F*_p_ = 2.35 J/cm^2^), the measured values were 31.0 ± 2.0 MPa (without thermal cycling) and 30.9 ± 2.0 MPa (after thermal cycling). Thus, there is no appreciable degradation upon a discrete and limited number of thermal cycles either for adhesive or cohesive joints. However, the important enhancement of bond strength by laser processing provides the required mechanical robustness of the joint, having notable advantages in terms of joint reliability.

Finally, it is worth noticing that the shear bond strength values obtained for the lap joints prepared after exposing the original copper surfaces to a chemical oxidizing agent for 3 or 5 min (as explained in Section 2.3) were 27.6 ± 3.1 MPa and 31.6 ± 2.0 MPa, respectively. 

### 3.5. Thermal Properties of Cu/Epoxy/Cu Joints 

Thermal properties of different Cu/epoxy/Cu joints were analyzed using the procedure described in Section 2.4. The purpose of the study is to analyze the main contributions to the thermal resistance of the joints and whether the observed differences in the copper/epoxy interfaces have a relevant effect on the thermal behavior of the joints. This study was focused on a laser-scanning processing method because of its higher interest for this particular application, as mentioned above.

**Figure 9:**
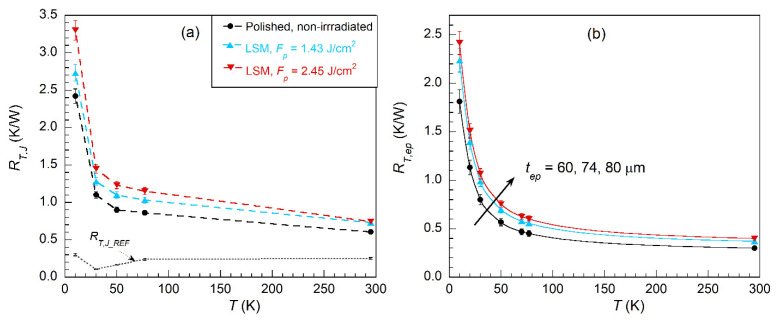
(**a**) Measured thermal resistance, *R_T,J_*, for three Cu/epoxy/Cu joints at different temperatures and $\dot{Q}$ = 0.5 W (Equation (1)). Contact area of the joint *A_J_* = 2 cm^2^. Copper surfaces of the joint were either polished or irradiated by laser using LSM method and given *F*_p_ values. (**b**) Estimated contribution of *R_T,ep_* (Equation (2)) taking into account the thickness of each joint.

Figure 9a shows the measured thermal resistance, *R_T,J_*, as a function of the temperature for the three Cu/epoxy/Cu joints displayed in Figure 6. For comparison purposes, the measured values of *R_T,J−REF_*, i.e., a similar joint but without epoxy, are also displayed in the figure. The temperature dependence of *R_T,J_* is similar for all three analyzed Cu/epoxy/Cu joints. The lowest *R_T,J_* values correspond to the joint derived from polished non-irradiated copper, while the highest correspond to the joint with copper irradiated at the highest pulse fluence (2.45 J/cm^2^, LSM). Figure 9b shows the thermal resistance contribution of the epoxy layer for each joint, derived from Equation (2) using *t_ep_* values measured from the SEM cross-section images. The error bars in Figure 9a take into account the standard deviation errors of *t_ep_* (4 μm). By comparing Figure 9a,b, it is observed that the temperature dependence of the thermal resistance of the joint is mainly determined by the epoxy layer thermal characteristics and thickness. 

Following the procedure explained in Section 2.4, the contribution of the epoxy/Cu thermal boundaries (*R_T,Cu−ep_*) can also be estimated. At room temperature, this value is 2·*R_T,Cu−ep_* ≈ 0.16 ± 0.03 K/W at 295 K, which is similar for the three measured joints within the estimated errors. However, at cryogenic temperatures, this contribution is higher for the joint made from irradiated copper at the highest pulse fluence (2.45 J/cm^2^). The thicker oxide layer and larger roughness of the copper surface of this sample results in a less clean copper/epoxy interface, affecting its thermal conductance properties. More in detail, the experimentally derived values for this joint are 2·*R_T,Cu−ep_* ≈ 0.4 ± 0.05 K/W and 0.7 ± 0.12 K/W at 50 and 10 K, respectively; meanwhile, for the other joints, 2·*R_T,Cu−ep_* ≈ 0.3 ± 0.05 K/W and 0.4 ± 0.12 K/W values are obtained at the same temperatures. Note that a thinner epoxy layer would produce a decrease of the thermal resistance of the joint, although due to the size of the alumina particles embedded in the epoxy, the minimum thickness would be limited to about 40–50 μm. 

From these results, it is clear that a proper selection of laser processing conditions of copper surfaces can achieve high shear bond strength (>30 MPa) with the possible lowest surface roughness and oxides, thus obtaining a good balance between mechanical bond strength and thermal characteristics. For the particular parameters used in this work, this would be obtained at *F*_p_ ≈ 2 J/cm^2^ (*I* ≈ 200 MW/cm^2^).

## 4. Conclusions

The effect of laser irradiation on copper surfaces on the mechanical and thermal resistance of Cu/epoxy/Cu has been analyzed in this work. Stycast-2850FT epoxy resin with catalysts 9 or 23LV was chosen for this study. Catalyst 9 has been found to be inadequate to obtain good bonding properties with copper surfaces, in contrast to catalyst 23LV. 

Shear bond strength of Cu/epoxy/Cu lap joints increases from about 17.0 MPa (adhesive failure) for non-irradiated polished copper surfaces, up to 30–32 MPa (cohesive failure) for optimum laser-processed samples. No significant time evolution has been found in the epoxy wettability nor in the mechanical properties of the Cu/epoxy/Cu joints prepared with laser-irradiated copper, in the time interval from a few minutes to about 22 days of this study.

Thermal resistance values of the Cu/epoxy/Cu joints at ambient and cryogenic temperatures have been found to be dominated by the properties and thickness of the epoxy layer. However, a certain deterioration of the thermal conductance of the copper/epoxy interface has been observed for samples treated under the most intense laser irradiation. Our results indicate that it is possible to obtain optimum laser processing conditions of the copper surfaces to achieve a good balance between the mechanical and thermal conductivity properties of Cu/epoxy/Cu joints. In consequence, laser technology can be considered an alternative to improve the adherence strength of these joints avoiding the use of chemicals to control the copper surface for their application both, at ambient and cryogenic temperatures. 

## Figures and Tables

**Table 1:** Oxygen content and apparent contact angles (water and epoxy) of Cu surfaces at given times after laser processing.

Laser Processing		Oxygen Content, at %		Water Contact Angle (°)			Epoxy Contact Angle (°)		
		Time < 1 h	22 Days	<1 h	1 Day	22 Days	<1 h	1 Day	22 Days
None		14.7			100			38	
LSM	*F*_p_ = 1.43 J/cm^2^	18.3	18.7	42	74	80	27	30	29
	*F*_p_ = 2.35 J/cm^2^	21.7	20.6	<10	55	95	32	27	30
Bit-map	150 dpi	17.5	25.1						
	300 dpi	20.6	27.4	<10	29	79	27	31	27
	600 dpi	27.4	33.3						

## Data Availability

Data available upon request from the authors.
